# Signaling to the Epigenome: New Insights into the Roles of Nuclear Signaling Kinases in the Context of the Immune System and Cancer

**DOI:** 10.3389/fimmu.2017.00980

**Published:** 2017-08-21

**Authors:** Pek Siew Lim, Kum Kum Khanna, Sudha Rao

**Affiliations:** ^1^University of Canberra, Canberra, ACT, Australia; ^2^QIMR Berghofer Medical Research Institute, Brisbane, QLD, Australia

**Keywords:** PKC, signaling kinase, epigenome, immune system, cancer, T cell, epigenetic regulation, transcription

**Editorial on the Research Topic**

**Signaling to the Epigenome: New Insights into the Roles of Nuclear Signaling Kinases in the Context of the Immune System and Cancer**

Transcriptional programs initiated by different external cues are tightly regulated by highly conserved signaling kinases. Though the action of signaling kinases is predominantly reported in the cytoplasm, emerging studies describe that their physiological and pathological functions are mediated through direct regulation of gene expression programs. The dual role of signaling kinases to regulate gene transcription occurs *via* cytoplasmic signaling to the nucleus, wherein they need to enter the nucleus to act as a novel class of epigenetic enzymes that directly tether to the chromatin template and exquisitely (or dynamically) regulate the epigenome.

The focus of this research topic is to highlight the current knowledge and understanding of nuclear signaling kinases in epigenetic regulation in the immune system and cancer. In particular, using T cells as a model system for immune gene regulation, we explore its role in cellular metabolism, T cell differentiation and activation, as well as its role in HIV regulation.

A key component of the immune response to external stimuli is the activation of the T cell receptor (TCR) signaling. Chisolm and Weinmann hones in on how transcription factors can influence T cell metabolic gene expression program to regulate T cell specialization. Specifically, TCR signaling activates transcriptional factors that control glycolysis, glutaminolysis, and lipid biosynthesis to regulate T cell differentiation into effector and memory T cells. The important concept of both effector T cells and cancer cells sharing similar transcription factors regulating cellular metabolism programs means an understanding of T cell proliferation coupled with metabolic programs could bring light to similar mechanisms in other cellular settings.

The regulation of gene expression in T cell differentiation occurs in the context of the chromatin structure. Nguyen et al. first introduce the mechanisms of modulating chromatin structure to control gene expression. Their review then focuses on the role of transcriptional enhancers to control how T cell identity is established and maintained. They highlighted how chromatin looping at interferon gamma loci and type 2 locus is linked to Th1 or Th2 differentiation in CD4+ T cells and also in CD8a expression in CD8+ T cell development and differentiation. Finally, the function of the enhancer in T cells at a genome-wide level was discussed from a normal and disease state.

One of the signaling kinases that are keys in T cell transcriptional response is the PKCθ. Brezar et al. provide an overall summary of PKCθ in regulatory and effector T cell functions and highlights the importance of PKCθ in the formation of the immunological synapse. Notably, PKCθ is known to positively regulate effector T cells but negatively regulate regulatory T cells. As the balance of these two T cell subsets is critical, the authors discuss examples of how dysregulation of PKCθ is linked to human diseases such as autoimmunity, cancer, and HIV. The knowledge gleaned from understanding the mechanisms of PKCθ regulation of T cell responses will be important in developing of PKCθ inhibitors as therapeutic targets against dysregulated immune responses.

In a subsequent paper, we are introduced to the role of PKCθ in CD4+ T cell development and HIV infection from Phetsouphanh and Kelleher. They briefly introduce PKCθ and expand on its role in T cell activation and T cell anergy. In terms of T cell activation, they highlighted recent findings on nuclear PKCθ and how it regulates inducible gene expression during normal T cell function and HIV transcription.

Lopez-Huertas et al. examined the mechanism in which PKCθ in conjunction with HIV-1 transcriptional regulator Tat can regulate HIV-1 replication in CD4+ T cells. They showed that PKCθ activity is required for activation of Ras/Raf/MEK/ERK signaling molecules and transcription factors such as NFKB. Furthermore, analysis at the nuclear chromatin level provided evidence for the coexistence and interaction of PKCθ and Tat at the HIV-1 LTR promoter.

McCuaig et al. provides further support by examining how PKCθ regulates alternative splicing in T cells. They studied SC35, a splicing factor in T cells, and found that it is not only induced upon viral infection in effector T cells but colocalizes with active histone marks. Furthermore, SC35 is shown to coexist with PKCθ, which directly phosphorylates SC35 at key regulatory domains, suggesting that nuclear PKCθ is a regulator of splicing factor SC35.

Signaling kinases act upon other transcriptional regulatory factors within the cell. In the nucleus, one key regulatory factor that is regulated by signaling kinases is transcription factors, which interacts dynamically with the epigenome. Critically, dysregulation of this dynamic interplay causes a disruption of the normal gene expression leading to the development of cancer. Brettingham-Moore et al. gives an example of the interplay of the transcription factors and the epigenome, with a particular focus on the RUNX1 transcription factor and how its oncogenic derivative RUNX1-ETO functions in leukemia. Whether RUNX1 behaves as a transcriptional activator or repressor is highly dependent on the epigenome context and occurs through the recruitment of transcriptional cofactors and epigenetic modifiers.

In addition to transcription factors, histone modifications are important in regulating gene expression and are actioned by histone modifiers, which can add or remove histone modifications from the histone tails. Casciello et al. describes one such histone modifier, G9a, a histone methyltransferase that methylates histone H3 lysine 9, and its role in cancer initiation and progression. Importantly, they present evidence of studies using small molecule inhibitors of G9a to treat cancers with some effectiveness in different cancer settings, suggesting G9a as a promising therapeutic target for cancer treatment.

Overall, it is critical to have a detailed understanding of these mechanisms of epigenetic regulation since the reversible nature of epigenetic aberrations makes them attractive targets for the clinical use of epigenetic therapies. While their cytoplasmic signaling functions are extensively studied, the new concept that these signaling kinases are regulating nuclear functions at the epigenome level is exciting and opens up a new area of research into deciphering the mechanism by which it works at a transcriptional level. It will be interesting to define the different roles of both cytoplasmic and nuclear signaling kinases and how these factors could be targeted together or separately for developing future therapeutic targets.

## Author Contributions

PL drafted and edited the manuscript. SR and KK edited the manuscript.

## Conflict of Interest Statement

The authors declare that the research was conducted in the absence of any commercial or financial relationships that could be construed as a potential conflict of interest.

